# Pregnancy in Cystic Fibrosis—Past, Present, and Future

**DOI:** 10.3390/jcm12041468

**Published:** 2023-02-12

**Authors:** Michal Gur, Mordechai Pollak, Ronen Bar-Yoseph, Lea Bentur

**Affiliations:** 1 Pediatric Pulmonary Institute and CF Center, Rappaport Children’s Hospital, Rambam Health Care Campus, Haifa 3109601, Israel; 2 Rappaport Faculty of Medicine, Technion–Israel Institute of Technology, Haifa 3525422, Israel

**Keywords:** cystic fibrosis, pregnancy, CFTR modulators

## Abstract

The introduction of mutation-specific therapy led to a revolution in cystic fibrosis (CF) care. These advances in CF therapies have changed the disease profile from a severe incurable disease with limited survival to a treatable disease with improved quality of life and survival into adulthood. CF patients are now able to plan their future, including marriage and parenthood. Side by side with the optimism, new issues and concerns are arising, including fertility and preparation for pregnancy, maternal and fetal care during pregnancy, and post-partum care. While cystic fibrosis transmembrane regulator (CFTR) modulators show promising results for improving CF lung disease, data on their safety in pregnancy are still limited. We performed a literature review on pregnancy in CF from the past, with the first described pregnancy in 1960, through the current fascinating changes in the era of CFTR modulators, to ongoing studies and future directions. Current advances in knowledge give hope for improved outcomes of pregnancy, towards the best possible prognosis for the mother and for the baby.

## 1. Introduction

Advances in the care of cystic fibrosis (CF) led to improved life expectancy. Thus, CF patients have been able to reach adulthood; consequently, issues like marriage and pregnancy became relevant. Clinicians are now required to address new challenges, such as fertility, family planning, pregnancy, and post-partum care [1].

The first pregnancy in CF was described in 1960, but unfortunately the mother deceased shortly after giving birth [2]. Thereafter, an increasing number of successful pregnancies were reported in the literature. According to the United States (US) CF patient registry, the yearly number of pregnancies increased from 230 in 2009 to 310 in 2019 [3]. This number nearly doubled in 2020, with a total of 619 pregnancies reported [4]. While in the past a cut-off of forced expiratory volume in one second (FEV1) > 60% was considered necessary to undergo a successful pregnancy, more recent studies report positive outcomes, even in women with advanced pulmonary disease. However, these women are at increased risk of premature delivery, and should be followed more closely [5]. 

In 2008, Edenborough et al. published guidelines for the management of pregnancy in women with CF. The authors addressed several important aspects, from the pre-conceptual until the post-partum period [6]. A few years later, the introduction of cystic fibrosis transmembrane regulator (CFTR) modulators opened new horizons for CF patients. Currently, approximately 90% of CF patients are eligible for this treatment, while the remaining 10% are still waiting for a novel precise medicine to address their molecular defect. The introduction of a new triple combination therapy of elexacaftor/tezacaftor/ivacaftor (ETI) in 2019 has been a significant game changer for eligible patients. Even in patients that were previously candidates for lung transplantation, the introduction of CFTR modulators resulted in such impressive improvement, that pregnancy has become feasible. In this context, new issues arise, such as the effect of CFTR modulators on fertility, and their safety during pregnancy [7]. It should be noted that mutation-specific therapy is still not available and funded for all eligible patients. 

The European Respiratory Society (ERS)/Thoracic Society of Australia and New Zealand (TSANZ) published a consensus statement about the management of pregnancy in CF, as well as other chronic airway diseases [8]. More recently, a multidisciplinary panel of women with CF and CF clinicians published recommendations on pre-conception, intrapartum, and post-partum care for people with CF [9].

Herein we performed a literature review on pregnancy in CF going over a longitudinal timeline: from the past—before the era of CFTR modulators; through present—current knowledge about factors affecting pregnancy outcomes, including the use of CFTR modulators in pregnancy; to the future—on-going studies and future directions for the care of pregnancy in women with CF.

## 2. Pregnancy before the Era of CFTR Modulators

### 2.1. Preparation for Pregnancy

In CF, as in other chronic diseases, there is utmost importance for optimization of pre-conception health; thus, discussion about fertility and family planning should begin during adolescent years and continue into adulthood [9]. One of the fundamental aspects of CF care is the multidisciplinary clinic. To prepare for pregnancy, all team members should be involved, targeting medical and psychological aspects, and optimizing chronic therapies, including inhalations of mucolytics and antibiotics, and chest physiotherapy [8]. In addition, it is important to hold a realistic discussion about the patient’s current health status, and the potential impact of pregnancy on maternal health and fetal outcomes, as well as short- and long-term morbidity [9,10]. 

#### 2.1.1. Infertility and Subfertility

Almost all adult men with CF are infertile due to failure of normal development of the vas deferens; although, there are functioning sperm in their testicles. Obliteration of the vas deferens by retained secretions in utero leads to congenital bilateral absence of vas deference (CBAVD) and azoospermia. Notably, CBAVD may be the sole manifestation of CF [3,11]. 

In women with CF, subfertility was first described in the 1970s. CFTR was found to be expressed in the cervical epithelium; thus, deficient CFTR led to thick cervical mucus and reduced sperm permeability [3,12]. In addition, malabsorption and chronic inflammatory state lead to impaired growth, and delays in puberty and menarche. After reaching menarche, anovulatory cycles have been an important cause of impaired fertility [12]. Girls with CF were found to have delays in achieving pubertal levels of insulin-like growth factor-I (IGF-I), follicle-stimulating hormone (FSH), and luteinizing hormone (LH) [13]; low levels of anti-Mullerian hormone (AMH), considered to be a marker of ovarian reserve, were found in women with CF [14]. 

In a retrospective study from 11 centers, a higher rate of subfertility was found in 605 CF woman (35%, compared to 5–15% in the general population). In a multivariate analysis, pancreatic insufficiency (PI) and older age were associated with subfertility, while lung function, body mass index (BMI), CF-related diabetes (CFRD), and number of exacerbations in the previous year were not associated. CFRD was present in 16% of women with normal fertility and 23% of sub-fertile women (*p* = 0.02) [15]. Similarly, a French registry study found a slightly higher rate of assisted conception in women with pre-gestational diabetes (53.8% vs. 34.5%, *p* = 0.06) [16]. In another study, a questionnaire was sent to women with CF, of whom 46 sought pregnancies; 17 (37%) had no spontaneous pregnancy, from whom 13 had infertility treatment and 11 were successful. 

#### 2.1.2. Genetic Testing

Women with CF who desire to get pregnant should undergo genotype analysis, if previously unknown. In addition, carrier screening should be offered for partners. As there are more than 300 disease-causing mutations in the CFTR gene, a limited panel of the 25 most common mutations may miss up to 30% of cases, especially in non-Caucasians. Thus, next-generation sequencing is the preferred method [12,17]. If the partner is found to carry a CF disease-causing mutation, pre-implantation genetic diagnosis may be offered [8]. While many developed and developing countries have implemented newborn screening for CF as routine, prenatal genetic screening tests are not broadly used. However, when there is family history of CF, and there is high suspicion that parents might be carriers, parental screening would be suggested. Ideally, genetic screening should be utilized for the suspected carrier and if a mutation is found, then the partner should be screened as well. These tests should be performed prior to conception in order to allow, for families that are interested, the possibility of pre-implantation genetic diagnosis (PGD). In our country, a unique population carrier screening (PCS) has been available since 1999, and universally subsidized since 2008. Stafler et al. assessed the impact of this screening program and showed a dramatic reduction in CF birth rates with a shift towards milder mutations [18]. 

#### 2.1.3. Risk Factors for Poor Outcome of Pregnancy in CF 

Several factors related to maternal health have been found to contribute to outcomes of pregnancy in CF. These include pancreatic insufficiency (PI) and nutritional status, CFRD, pulmonary and cardiac function, and bacterial burden [17].

Nutrition should be optimized through diet, fat-soluble vitamins, and pancreatic enzyme replacement therapy (PERT). Poor nutritional status has been found to be related to poor outcomes, including prematurity and low birth weight [17]. A BMI of 22 kg/m^2^ is considered the goal prior to pregnancy [9]. Historically, weight gain of 11 kg (kg) in pregnancy was recommended; recent recommendations depend on pre-pregnancy BMI, with higher weight gain expected for women with low BMI [17]. 

CFRD was also found to be a risk factor for poor outcome of pregnancy. Adequate glucose control should be achieved prior to and during pregnancy, with a goal of hemoglobin A1c (HbA1c) of <6.5% at the time of conception and <6.0% during pregnancy. Gestational diabetes is more common in women with CF, occurring in 10–36% of pregnancies [9]. Guidelines for CFRD in pregnancy recommend an oral glucose tolerance test (OGTT) at 12–16 weeks and at 24–28 weeks of pregnancy to detect the development of gestational diabetes [19]. 

In patients with low pre-conception BMI, or poor weight gain during pregnancy, enteral feeding should be considered. However, enteral tube feeding may unmask diabetes, thus closer follow up and blood glucose monitoring are warranted [10]. 

Several studies suggest that cor pulmonale and pulmonary hypertension are absolute contraindications for pregnancy in CF women. Increased right ventricular pressure can cause right ventricular dysfunction, tricuspid regurgitation, and, in severe cases, death. The mortality in parturient with pulmonary hypertension is reported to be around 30%. Thus, screening patients with CF with an echocardiogram during prenatal care is very important [20].

### 2.2. Therapies during Pregnancy

The treatment regimen for CF patients is often complex, and includes PERT, antibiotics (inhaled or oral), mucolytics, and anti-inflammatory medications [12]. During pregnancy, the therapeutic benefits for the mother, possible adverse effects on the fetus, and the risks of cessation of therapies, all should be considered [9,21]. Because pregnancy is almost always an exclusion criterion in clinical trials of new therapies, much of the available data are based on animal models rather than human studies [9]. This illustrates the importance of an individualized approach in optimizing CF care during pregnancy, specifically for those with more compromised lung function. During the first six months of pregnancy, the woman should be seen frequently in clinic, perhaps monthly and two-weekly in the final trimester or more frequently as progress dictates. At each visit, physical examination, measurement of oxygen saturation and pulmonary function, and weight should be performed, and sputum should be obtained [6]. 

To ensure adequate nutritional status and normal vitamin levels, PERT and vitamin supplementation should continue during pregnancy [8]. PERT appears to pose no significant risk during pregnancy. Vitamin A should be given in doses of less than 10,000 IU daily; higher doses in early pregnancy have been found to be associated with neural crest defects [12,17]. Folate supplementation is recommended as in non-CF pregnancy guidelines [9]. 

The two most common inhaled mucolytics are dornase alfa and hypertonic saline; both are used as chronic maintenance therapies. Both are considered safe to use in pregnancy, as the systemic absorption is minimal [9]. 

Airway clearance therapy (ACT) and physical activity should be established as routine before pregnancy and continued through pregnancy. As reflux may be increased during pregnancy, adjustment of physiotherapy may be necessary; timing of physiotherapy before meals, as well as upright sitting during ACT, may aid in minimizing reflux [8]. In severely ill patients, supplemental oxygen or even non-invasive assisted ventilatory support may be required during physiotherapy. If oxygen saturation falls to below 90% during ACT or physical exercise, supplemental oxygen should be administered to maintain oxygen saturations at around 92%. Overnight pulse-oximetry should be considered to check for nocturnal desaturation [6]. 

Inhaled antibiotics (AB) are recommended for patients chronically colonized with Pseudomonas aeruginosa (PSA). Tobramycin, aztreonam, and colistimethate sodium are most used. As in mucolytics, the inhaled route is considered safe due to minimal systemic absorption [9]. 

Azithromycin is a macrolide oral AB used as maintenance therapy due to its immunomodulatory effects. The drug is considered “probably safe” to continue during pregnancy; while women should be informed about a very small potential risk for the fetus, it is important to note that the risk of cessation of treatment is unknown [9,17]. It should be noted that treatment with azithromycin in the neonatal period was found to be associated with pyloric stenosis [22].

#### Treatment of Pulmonary Exacerbations (PEx)

The general approach for treating PEx in CF includes optimizing ACT, as well as the use or oral or intravenous (IV) AB, targeting the most recent cultured respiratory pathogens. Mild PEx usually is treated by oral AB, while moderate to severe exacerbations require IV treatment. Systemic AB use in pregnancy may be challenging due to placental transfer and potential risk of teratogenesis of some antibiotics [9]. 

Staphylococcus aureus (*S. aureus*) and PSA are the two most prevalent pathogens in the airways of CF patients. For the treatment of S. aureus, penicillins and cephalosporins are considered safe to use throughout pregnancy [9,12]. Trimethoprim-sulfamethoxazole use in the first and third trimesters has been associated with neural tube defects and hemolytic anemia, respectively. Therefore, their use should be avoided during the first and third trimesters, and at delivery [9,23]. 

Treatment of methicillin-resistant *S. aureus* (MRSA) in non-pregnant CF patients includes trimethoprim/sulfamethoxazole, vancomycin, or linezolid. Human studies of vancomycin are limited to first trimester, but no teratogenic effect has been found. Thus, vancomycin is considered “probably safe”. Regarding linezolid, animal models have not shown a teratogenic effect, but data in humans are limited to case reports. Thus, it should be used if allergies or intolerance prevent the use of vancomycin, and only if benefit outweighs the risk [9].

For the treatment of PSA, the fluoroquinolones (levofloxacin and ciprofloxacin) are the oral AB of choice. Due to concerns for cartilage damage in animal models, their use has been typically avoided in pregnancy. However, recent evidence in human studies suggests low risk of teratogenesis, and they are considered “possibly safe” [8,9]. If a fluoroquinolone is indicated, ciprofloxacin is probably the preferred choice [6]. Anti-pseudomonas beta-lactam antibiotics (ceftazidime, ticarcillin) are considered safe during pregnancy. IV aminoglycosides can potentially cause fetal nephrotoxicity and ototoxicity, and their use should be limited to critically ill patients. If given during pregnancy, the once-daily dosing is preferable, with monitoring of drug levels [9,10,23]. Meropenem is considered “possibly safe” during the first trimester and “probably safe” during the remainder of pregnancy. However, women with CF have been found to be at increased risk of pre-eclampsia, and carbapenems lower the seizure threshold [9]. 

Table 1 summarizes the recommendations for CF therapies during pregnancy.

### 2.3. Outcome of Pregnancy

CF team, obstetricians, anesthesiologists, and the patient should be prepared for the delivery. Most CF women can have a spontaneous delivery with careful hemodynamic monitoring. Cesarean delivery should be reserved for usual obstetric indications. The increased cardiac output during delivery (about 50%) poses an acute risk of heart failure in women with pulmonary hypertension or core pulmonale. Regional analgesia may be beneficial for women with CF as it provides ability to rest and increased pain control [17].

In most studies, approximately two-thirds of births to women with CF are by vaginal delivery. Previous studies found an increased rate of cesarean section (CS) in preterm deliveries and in women with lower pulmonary function or CFRD. In cases of CS for maternal or fetal indications, spinal anesthesia is preferred over general anesthesia. After delivery, adapted analgesia is important to reduce pain, fatigue, and anxiety, and to allow re-initiation of airway clearance soon after delivery [3].

The use of extracorporeal membrane oxygenation (ECMO) during pregnancy is described in an interesting systemic review by Naoum et al. Some of the reported patients were diagnosed with pulmonary hypertension [24]. However, data on use of ECMO for the obstetric population with CF are extremely limited. One unique case of perioperative management and preemptive ECMO cannulation in a parturient with CF undergoing CS was reported by Dos Santos et al. and highlights individualized approach for CF pregnancy and delivery [20].

Most CF pregnancies result in live births, ranging from 67% to 85% in series from the United States, Canada, United Kingdom, and France [12]. As already mentioned, poor nutritional status and CFRD are considered as risk factors for poor pregnancy outcomes. Low baseline weight is associated with prematurity and low birth weight. Pre-conception hyperglycemia is associated with a higher risk of fetal malformations, while hyperglycemia during pregnancy is associated with fetal macrosomia and increased risk of caesarean section (CS) [9]. 

Pre-pregnancy lung function is often considered as the most important factor for predicting pregnancy, both for the mother and the baby. Women with poorer lung function at the beginning of pregnancy have a higher risk for prematurity or small-for-gestational age (SGA) baby [17,25]. Case series have shown increased risk of miscarriages, both early and mid-pregnancy, especially in mothers with worse lung function. Thus, some authors have suggested that a pre-pregnancy FEV1 >60% predicted should be present before pregnancy is undertaken [8]; however, there are reports of successful pregnancies, even in advanced lung disease. In general, Cheng et al. found that birth weight was lower with maternal FEV1 < 50% [26]. In a French survey, 61/77 (79%) pregnancies resulted in live births, with 19% and 16% of premature deliveries and low birth weight, respectively; the rate of CS was 16% [27]. In a small Scandinavian series, the rate of preterm delivery was 24%; low maternal weight and poor maternal lung function before pregnancy increased the risk of preterm delivery [28]. More recently, Reynaud et al. found that in women with FEV1 < 50%, the rate of CS was higher and birth weight was lower, but rate of prematurity was similar [5]. Similarly, the Toronto CF database reported a low rate of prematurity, with similar rates in those with FEV1 above and below 50% [29]. In a population-based UK study, 69/71 (97.2%) pregnancies were live births. Similar to previous findings, maternal FEV1 correlated with gestational age and birth weight [25]. Recently, in a multicenter-retrospective cohort study, moderate–severe disease (FEV1 ≤ 60% and/or BMI ≤ 21 kg/m^2^ prior to pregnancy) was associated with prematurity, while pancreatic insufficiency (PI) increased the risk for SGA infants [30]. 

#### Effect of Pregnancy on Lung Disease and Overall Survival

In 1995, Edenborough et al. followed 22 pregnancies in 20 women and found a 13% decrease in FEV1 during pregnancy [31]. A few years later, they performed a multi-center study with 72 pregnancies in 55 patients, and 65% of them required IV AB treatment during pregnancy [32]. Data from a French registry found even worse outcomes: 77.5% of women required IV AB treatment. Maternal mortality within one year after birth was 15% for those with FEV1 < 50% and 3% for those with FEV1 > 50% [33]. Nevertheless, other studies reported better outcomes. In the Toronto database mentioned earlier, pregnancy did not result in a greater decrease in FEV1 on follow up of 11 years. Nineteen percent of women died: FEV1 < 50%, colonization with Burkholderia cepacia, and PI were associated with decreased survival [29]. It should be noted that these studies followed pregnancies more than 20 years ago. The French registry data are from 1980–1999, and the Toronto database is from 1961–1998. Some authors even found that pregnancy was not associated with decreased lung function or shortened survival [1,34]. 

Data from the CF Epidemiologic Study demonstrated increased AB use during pregnancy, as well as increased outpatient and inpatient hospital visits [35]. Similarly, in the retrospective study mentioned earlier, patients with FEV1 ≤ 60% and/or low BMI ≤ 21 kg/m^2^ experienced more disease related complications during and after pregnancy; but on the other hand, FEV1 and BMI did not decline more rapidly after pregnancy [30]. 

In a large cohort study, women who reported pregnancy (*n* = 680) were more likely to have better baseline FEV1. Using Kaplan–Meier survival curves, the 10-year survival rate in pregnant women was higher than in those women who did not become pregnant (77% (95% confidence interval (CI), 71 to 82%) compared to 58% (95% CI, 55 to 62%), respectively). The pregnant cohort also had better survival among specific high-risk subgroups, FEV1 < 40% (*p* < 0.001) and CFRD (*p* = 0.02). The authors postulated that closer follow up during preparation for pregnancy resulted in improved compliance and better baseline health status [34]. 

Pregnancy was also found to increase the risk of diabetes. The number of women receiving diabetes therapy more than doubled during pregnancy, and half of them required long-term therapy [1]. In the French study mentioned earlier, there was no difference in FEV1 and BMI decline following pregnancy between those with and without CFRD [16]. 

### 2.4. Pregnancy after Lung Transplantation (LTX)

In 2006, Gyi et al. described 10 pregnancies in lung transplant recipients, and demonstrated that pregnancy after LTX is feasible. There were nine live births, five of them premature. Three women developed rejection during pregnancy, and four died within 38 months after delivery [36].

In a registry analysis from the US National Transplantation Pregnancy Registry, 12/30 patients had CF. There were 60% preterm births, with 11 infant complications and two neonatal deaths. The rate of rejection was higher in CF patients (25% vs. 11%), and mean birth weight was lower [37]. 

Some lung transplant centers recommend avoiding pregnancy all together; others recommend waiting for 2–3 years after LTX. Patients with a history of acute or chronic rejection should be advised against pregnancy [12,36]. 

Caution should be also taken with immunosuppressive medication use during pregnancy. Therapies such as cyclosporine and tacrolimus require frequent monitoring, and dose adjustment with weight changes. Mycophenolate mofetil is teratogenic and should be avoided. Safety data on other immunosuppressants, such as prednisone, azathioprine, and calcineurin inhibitors, remains limited [17]. 

Overall, pregnancy post lung transplantation is still considered high risk. Patients should be carefully counselled about the potential risks to graft function and overall health. 

**Figure 1:**
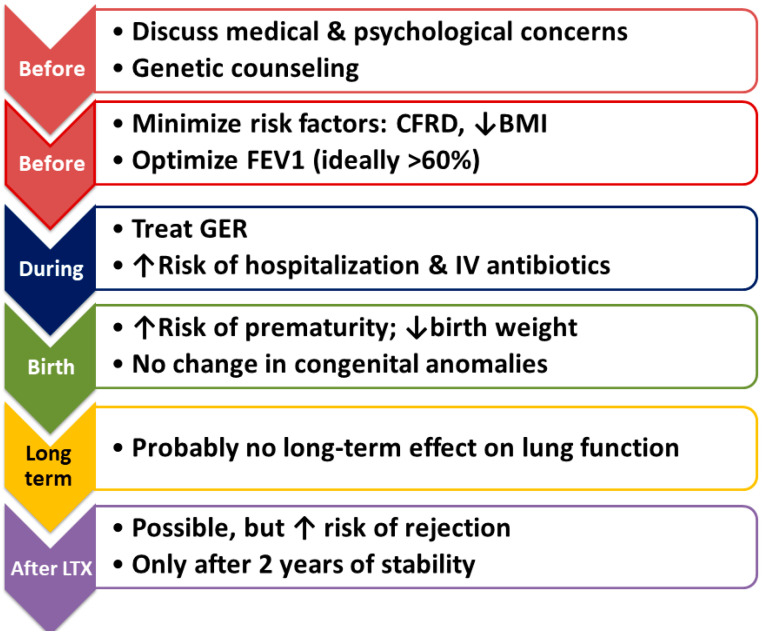
Summary of recommendations pre-conception, during pregnancy, and post-partum. CFRD = cystic fibrosis-related diabetes; BMI = body mass index; FEV1 = forced expiratory volume in one second; GER = gastro-esophageal reflux; IV = intravenous; LTX = lung transplantation.

Figure 1 summarizes the considerations and recommendations before pregnancy, during pregnancy, and in the post-partum period, including after LTX. 

## 3. Pregnancy in the Era of CFTR Modulators

### 3.1. Introduction

Three decades after the discovery of the CFTR gene [38], mutation specific therapy is now available for most CF patients. Ivacaftor was the first mutation-specific therapy approved for clinical use and improved FEV1 by an average of 10.6% for those with the G551D gating mutation [39,40]. Shortly after, two modulator combinations were shown to modestly improve FEV1 and decrease pulmonary exacerbations [41,42,43]. Finally, in 2019, trikafta, a new triple combination therapy of elexacaftor/tezacaftor/ivacaftor (ETI) showed extraordinary results. ETI improved FEV1 by an average of 13.8% in eligible patients [44,45]. This combination therapy has changed the lives of patients who are eligible for it, which is estimated to be 90% of CF patients. As mentioned earlier, one of the most exciting consequences of the new CF era is that people that were previously preoccupied with health and survival issues are now healthy enough and emotionally ready to consider planning families and children of their own. In this section we will focus on the impact of CFTR mutation-specific therapy on reproductive health in CF. 

### 3.2. Effects of CFTR Modulators on Fertility

Since development of the vas deferens is already disturbed in utero, eventually leading to obstructive azoospermia, it is unlikely that CFTR modulators will have a significant impact on this aspect once fetal development is completed. However, an interesting study found that in CF ferrets homozygous for the gating mutation G551D, treatment with ivacaftor during pregnancy led to development of the vas deferens and epididymis [46]. Whether it is possible to rescue the formation of vas deferens following in utero exposure to highly efficient modulators such as ETI, and whether it is safe, is still unknown. Additionally, males with CF are known to have smaller volume of ejaculate than healthy men. There is a possibility that CFTR modulators can lead to increased ejaculate volume by acting on CFTR channels present in the male reproductive tract [47]. Perhaps related to that, some cases of acute testicular pain in men with CF have been reported following initiation of ETI therapy. The pain onset occurred within two weeks of ETI initiation and in all cases but one, the symptom resolved within one week [48]. While the mechanism of this side effect is still unknown, it can be speculated that restoration of CFTR function in the male reproductive tract had a role in this side effect. 

As mentioned earlier, the reproductive anatomy in women with CF is not different than healthy women, but several factors contribute to subfertility. Mutation-specific therapy may potentially increase fertility rates, both by directly affecting the female reproductive tract, and indirectly by improving nutrition and health status. 

Although it is early to appreciate the impact of ETI on fertility fully and accurately, experience with ivacaftor showed improved fertility in women that were previously considered infertile. In the phase 3 study of ivacaftor, despite the fact that women agreed to use contraceptives while on the study, 2% of the women became pregnant, thus illustrating the importance of proper guidance for women starting use of CFTR modulators [39,49,50]. The exact mechanism of improved fertility is not yet known, but the CFTR modulators are thought to decrease viscosity and increase pH in cervical mucous secretions, promoting a more fertile environment [49].

### 3.3. Use and Safety of CFTR Modulators in Pregnancy

Placental transfer of CFTR modulators has been observed in animal models and in humans [51,52]. When tested in three women using ETI during pregnancy, ETI was found in high concentrations in cord blood, suggesting that concentrations in utero are within therapeutic range. Although in lower levels, detectable concentrations of ETI were also seen in breast milk [21]. In line with that, an astonishing case report described a CF patient homozygous for F508del mutation, treated with ETI before and during pregnancy. The fetus was found to have echogenic bowel on week 20 and was found to be F508del homozygous as well. The mother chose to continue ETI despite the lack of sufficient safety data. At week 32, the fetal bowel was reported to be normal. Finally, the baby was delivered at term, with no meconium ileus. Surprisingly, the baby had normal immunoreactive trypsinogen (IRT) levels, leading to a false negative newborn screening for CF. Moreover, despite having typical CF, he did not suffer from PI. Repeated tests showed normal fecal elastase (>200 µg/g), and therefore PERT was stopped [21]. In another similar case, a F508del carrier was pregnant with a F508del homozygous fetus. The fetus was diagnosed with meconium ileus at 23 weeks of gestation; thus, the mother began ETI at 32 weeks with intent to treat fetal meconium ileus. A female infant was delivered at 36 weeks with no complications. Fecal elastase at age two weeks was 240 mcg/g and maternal and infant liver enzymes were within normal levels [53].

In terms of safety, when tested on animal models, elexacaftor and ivacaftor given at high doses that are toxic to humans, caused impaired fertility in male and female rats. However, CFTR modulators did not show adverse impact on fertility or show teratogenicity when given at normal human doses [54]. Another area of concern is the development of bilateral cataracts. Toxicity studies of ivacaftor in rats demonstrated infant cataracts, but until recently this was not reported in human infants. Recently, Jain et al. described three cases of infants exposed to ETI in utero and while breastfeeding, who were found to have bilateral congenital cataracts within six months of birth. Importantly, none of these infants had significant visual impairment [55]. 

### 3.4. Pregnancy Outcomes with CFTR Modulators

Currently, there is a lack of high-quality data to properly assess the maternal and fetal effects of CFTR modulators given during pregnancy. Until today, some case reports and surveys have been published, giving first signs of optimism in this area. 

In a recent review by Taylor-Cousar, several case reports of women using ivacaftor or lumacaftor/ivacaftor were summarized. Out of 11 cases described, no adverse impacts on the infants were reported (other than prematurity that was attributed to significant maternal lung disease). One infant was reported to have mild hyperbilirubinemia that resolved spontaneously. In four cases, a formal ophthalmologic exam was carried out to the fetus, and all exams were normal [54]. Interestingly, there have also been reports of patients that stopped CFTR modulators due to concerns about the unknown safety to their fetus, and eventually re-introduced therapy due to declining lung function [56]. 

On a larger scale, two international surveys were reported on the outcomes of pregnancies for women treated with CFTR modulators. Their results are summarized in Table 2. The first survey focused on outcomes of pregnancies prior to ETI. A total of 64 pregnancies in 61 women were reported. Of these, 31 pregnancies were exposed to ivacaftor, 26 to lumacaftor/ivacaftor, and 7 to tezacaftor/ivacaftor. The physicians were asked to report their opinions about whether the complications were related to treatment. Only two complications were reported as being related to modulator use—one pulmonary exacerbation, and a case of acute myelocytic leukemia (AML). Notably, no other reports have shown a connection between leukemia with use of CFTR modulators. On the other hand, in those choosing to stop therapy due to unknown risks to the fetus, nine women experienced a decline in lung function leading to re-introducing modulator use during pregnancy [57]. 

A similar, more recent survey, using the same methodology, focused on outcomes of pregnancies for those treated with ETI [58]. In this survey, 47 pregnancies in 46 women were reported. Forty-one women were on ETI at the time of diagnosis of pregnancy and six elected to discontinue after pregnancy was diagnosed. Four women decided to begin ETI in the second or third trimester. Twenty-three women continued ETI throughout all trimesters. There were four miscarriages reported in the first trimester (8.9%); none was classified as related to ETI therapy (in one, the relation was deemed unknown, and the rest non-related). As reported in older CFTR modulators and perhaps to a larger extent, five women that elected to stop ETI, experienced a deterioration in lung function or increased symptoms; one patient experienced a severe deterioration including massive hemoptysis and significant fall in lung function. In terms of maternal outcomes, 28 events were deemed unrelated to therapy, one event was thought to be related to therapy (cholecystitis requiring cholecystectomy), and two with unknown relationship to ETI (cholestasis). Results of formal cataract inspections were not available in this report. At last, two cases were reported of unintended pregnancies shortly after delivery, and eventually were terminated [58]. The current limited data call for careful consideration of the beneficial versus the toxic effects of mutation specific therapy during pregnancy and lactation and continuing data collection and analysis. 

According to the US prescribing information (USPI) of ETI, there are limited and incomplete human data from clinical trials on the use of ETI or its individual components in pregnant women to inform a drug-associated risk [59]. Therefore, there is no assigned FDA risk category [60]. According to the European drug information, as a precautionary measure, it is preferable to avoid the use of ETI during pregnancy [61]. 

For infants exposed to ETI during pregnancy or breastfeeding, it is recommended to perform ophthalmologic screening examinations for neonatal cataract evaluation and liver function testing [9].

## 4. Conclusions and Future Directions

In conclusion, optimizing pre-conception medical and emotional status, addressing specific risk factors that are associated with poor outcome; integrated multi-disciplinary care during pregnancy, taking in account the risk-benefit ratio of additional treatments; and close follow up in the post-partum period, are all necessary for the best outcomes of pregnancy in CF patients, both for the mother and the newborn. 

As detailed above, at this point, data on the safety of CFTR modulators in pregnancy are limited. There is some evidence, however, that cessation of therapy may lead to declining lung function or increasing symptoms. On the other hand, there is no evidence for significant teratogenicity or severe side effects related to pregnancy. Accordingly, a European Respiratory Society/Thoracic Society of Australia and New Zealand statement concerning the management of reproduction and pregnancy in women with airway diseases, considered CFTR modulators to be “probably safe”, and that maternal benefit may outweigh potential risk during pregnancy and/or breastfeeding [8]. 

As discussed, modulators have been proven to cross the placenta efficiently, and limited evidence shows that maternal treatment can potentially rescue some of the irreversible damage that occurs in utero. Additional safety and efficacy data are still needed, but perhaps the dream may come into reality in the near future. Prenatally treating a fetus that is known to have CF may change the course of CF; we may postulate with caution, that even areas previously considered untreatable, such as pancreatic function and male fertility, may be reversible. 

Currently, the “Maternal and Fetal Outcomes in the Era of Modulators” (MAYFLOWERS) study sponsored by the CF Foundation and CF Therapeutics Development Network, is taking place in 40 CF care centers across the United States. CF women on CFTR modulators are enrolled during the first trimester of pregnancy to assess maternal and fetal outcomes during and after pregnancy [62]. 

Until additional information is available and specific guidelines are published, the decisions should be individualized and a risk–benefit approach should be discussed to ensure the best combined maternal and fetal health.

## Figures and Tables

**Table 1:** Summary of recommendations for CF therapies during pregnancy.

Drug	Use in Pregnancy	Remarks
PERT	Probably safe	
DEKAs	Probably safe	Vitamin A doses < 10,000 IU
Dornase alfa	Probably safe	Inhaled route—minimal systemic absorption
Hypertonic saline	Probably safe	
ACT	Continue during pregnancy	Adjust to minimize reflux
**Antibiotics**		
Inhaled AB	Probably safe	Minimal systemic absorption
Inhaled levofloxacin	Probably safe	Minimal systemic absorption
Azithromycin	Probably safe	Minimal possible risk for fetus
Penicillins and cephalosporins *	Probably safe	
TMX/SMZ	Avoid 1st and 3rd trimester and delivery	Neural tube defects, hem. anemia
Vancomycin	Probably safe	
Linezolid	No harm in animal models	Limited human data
Ciprofloxacin	Possibly safe	Concern of cartilage damage
Levofloxacin	Possibly safe	Ciprofloxacin is preferrable
Aminoglycosides *	Save for critically ill	Nephrotoxicity & ototoxicity
Meropenem *	Possibly safe in 1st trimester	Lowers seizure threshold

PERT = pancreatic enzyme replacement therapy; DEKAs = vitamin D, E, K, A (fat-soluble vitamins); AB = antibiotics; ACT = airway clearance therapy; TMX/SMZ = trimethoprim-sulfamethoxazole; * = intravenous (IV).

**Table 2:** Use of CFTR modulators in pregnancy according to international surveys.

Modulator	Pregnancies/Modulator Used throughout Preg, (*n*)	Miscarriage	Prematurity	Fetal Complications		Maternal Complications	
				Related to Modulator *	Unknown/Not Related	Related to Modulator *	Unknown/Not Related
**IVA**	31/15	2	-	0	3	0	16
**LUM/IVA**	26/16	0	4	0	8	2	17
**TEZ/IVA**	7/5	1	-	0	2	0	5
ETI **	47/23	4	5	0	20 ^a^	1	30 ^b^

IVA = ivacaftor; LUM = lumacaftor; TEZ = tezacaftor; ETI = elexacaftor + tezacaftor + ivacaftor; preg. = pregnancy; *n* = number. * Determined by a CF specialist responding to the survey. ^a^ None of the fetal complications were considered related to ETI. The most common complication was cesarean section (*n* = 4) as a result of infant factors (large for gestational age, *n* = 2; abnormal presentation, *n* = 2). Three infants were born with mild congenital malformations, for whom the relatedness was considered unknown; two of them were born to mothers with poorly controlled diabetes. One fetus had multiple anomalies (considered unrelated to ETI) and pregnancy was terminated. One patient had trisomy 16. ^b^ Events that occurred in more than one woman—gestational diabetes (*n* = 2), preeclampsia (*n* = 2), pre-term labor (*n* = 2), and need for Cesarean section (C-section) for maternal reasons (*n* = 4); two seizures occurred in a woman with a history of seizures. All these were considered unrelated to ETI. There were three events of cholestasis—one cholecystitis considered related to ETI, one episode in a woman with previous obstetric was considered unrelated, and one was considered with unknown relatedness. ** Recently, Jain et al. described three cases of infants exposed to ETI in utero and while breastfeeding: bilateral congenital cataracts were found, with no significant visual impairment [55]. Nash et al. for IVA, LUM/IVA and TEZ/IVA [54], Taylor-Cousar et al. for ETI [55].

## Data Availability

The data presented in this study are available on request from the corresponding author.
